# Health first, for all: Envisioning a novel complementary pathway for translational research

**DOI:** 10.1017/cts.2023.613

**Published:** 2023-08-18

**Authors:** Kevin Fiscella, Reza Yousefi Nooraie

**Affiliations:** 1 Department of Family Medicine, University of Rochester, Rochester, NY, USA; 2 Department of Public Health Sciences, University of Rochester, Rochester, NY, USA

**Keywords:** Health equity, population health, translational research, determinants of health, implementation research

Prior to the COVID-19 pandemic, US life expectancy was declining [1]. Psychological distress has risen over the past decades [2]. Among high-income countries, US life expectancy declined the most during the pandemic [3]. Scientific innovation in the USA seems to be slowing [4]. The current biomedical translational pathway alone may be insufficient for improving population health and health equity in the USA [5]. The NIH strategic plan identifies “Health Promotion and Disease Prevention” among its top three research priorities [6]. To address this national crisis, we propose a novel, complementary pathway for translational research: “Health First, for All.”

## The Biomedical Research Translational Pathway Benefits and Limits

NIH’s core mission is biomedical research, with more than half of its budget spent on basic biomedical and behavioral research [7]. Biomedical research has yielded groundbreaking successes – mapping the human genome, discovery of the gene-editing tool CRISPR, and novel treatments for viral infections and various cancers.

Yet, there are fundamental limits to the biomedical pathway. First, its basic, disease-based model of health is limited in scope because it ignores the positive aspects of health that are important to people and communities, including agency, resilience, adaptability, and well-being [8]. It misses opportunities for discovering how to optimize all people’s potential for health, particularly by addressing behavioral, environmental, and social determinants of health. Second, it is not optimized for improving health because it begins with the discovery of biomedical mechanisms that may or may not ultimately translate into population health benefits over its 17-year time course. Last, the biomedical pathway often ends with FDA approval, failing to cross “the second valley” of death, that is, adoption of the innovation for the benefit of all people [9].

## A Health First, for All Pathway for Translational Research

We propose a novel, complementary pathway based on three principles. First, this research pathway should start with the end goal of promoting health for all (rather than basic discovery relevant to diseases) and target health determinants (rather than mechanisms). Second, this pathway should begin with research on optimal strategies for addressing health determinants, particularly human material and psychological needs, that is, autonomy, competence, and relatedness [10], that promote well-being and capability for health with an equity lens. This research should be conducted in full partnership with residents of communities, particularly those marginalized, starting with community priorities for health and corresponding determinants, aiming for meaningful and sustainable engagement and co-ownership [11]. Third, this complementary pathway should be based on long-term partnerships with potential intervention adopters including communities and healthcare based on team science principles [12]. Identifying and addressing behavioral (e.g., physical activity, nutrition, mental health, etc.), environmental, and social determinants of health requires long-term, multisectoral funding, co-led community partnerships, cross-training among partnership members, and the application of equity-sensitized implementation science to communities and healthcare organizations. This requires genuine co-ownership of the process beginning with identifying priorities related to determinants of health and beginning by generating new evidence and adapting current evidence-based interventions to address community-identified priorities. When interventions are lacking, these community–research partnerships can activate “reverse translation” pathways to inform efficacy or even basic studies to develop new interventions [13]. Conversely, when barriers to translation exist, dissemination and implementation research can address implementation barriers at various levels including health-related policies [14,15].

## Implementing a Health First, for All Research Translational Pathway

**Figure 1.**
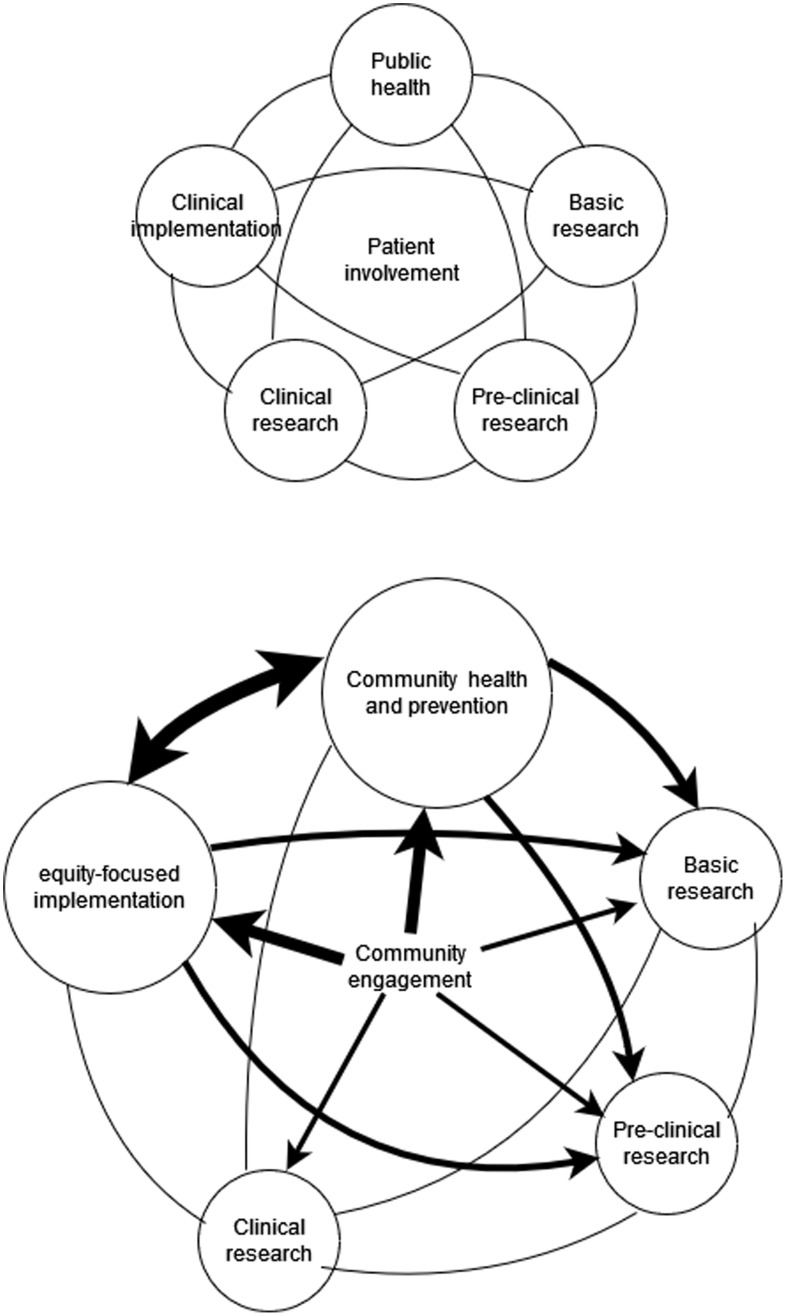
Current translational pathway (upper diagram) and proposed complementary Health First, for All pathway (lower diagram).

Implementation of a second translational pathway that interdigitates with the biomedical pathway is daunting (Fig. 1). To start, a national definition of health is needed beyond the absence of disease that acknowledges the fundamental human aspects of health [5]. Creating such a definition should involve a participatory process that includes federal departments and agencies working with communities and informed by the National Academies (NASEM) report on achieving whole health [16].

A long-term research agenda coupled with appropriate funding is needed for promoting and optimizing health. Establishing this agenda should begin with an NIH strategic plan in conjunction with diverse and marginalized communities. Potentially, the NCATS with additional funding could support this second “Health First, for All” translational pathway. This research agenda is urgently needed to inform the implementation of NASEM’s and VA’s whole health model and related value-based payment models [16,17].

Last, there is a need to cross the “second valley of death” with long-term funding for infrastructure and training required for partnerships between researchers, communities, and healthcare organizations to create a context for generating scientific knowledge on pragmatic strategies to address population health determinants. These co-led research–community–healthcare partnerships will require new models for collaboration and new delivery models for generating new evidence (with emphasis on appropriateness and diversity of perspectives and priorities) and testing strategies for the adoption of evidence-based, culturally adapted interventions [18]. These tripartite partnerships must begin with local health priorities and chosen evidence-based interventions and involve adaptations to local needs, implementation of strategies based on context, adoption of steps to ensure sustainability, methodologically rigorous assessments of health impact and equitable implementation processes. One potential approach to organizing programs across these entities around health determinants is to scale models like the NIH Compass program [19].

## Potential Benefits

Our national health is in precipitous decline [1]. The current biomedical translational research pathway is insufficient to reverse this crisis. We urgently need a second, synergistic rescue pathway. Starting with communities’ desired health outcomes and conducting collaborative research on how to optimize the adoption of current evidence for the benefit of all could shorten the time needed to improve population health. By prioritizing elements of health and health promotion that are most impactful and meaningful to people and their communities, this approach offers the potential for improving health in ways that matter most to people.

Roughly 90% of healthcare spending in the USA goes to people with chronic physical and mental conditions [20]. A “Health First, for All” pathway offers the potential to interrupt this vicious cycle by generating scientific knowledge to guide the optimization of individual and community health and well-being while addressing key determinants that yield inequities in health. Doing so could forestall chronic disease and ultimately slow unstainable growth in healthcare spending. Most importantly, this second translational pathway is critical to reversing our national decline in health and addressing long-standing health equities.
